# Review: Hippocampal sclerosis in epilepsy: a neuropathology review

**DOI:** 10.1111/nan.12150

**Published:** 2014-07-01

**Authors:** Maria Thom

**Affiliations:** Departments of Neuropathology and Clinical and Experimental Epilepsy, Institute of Neurology, University College LondonLondon, UK

**Keywords:** hippocampal sclerosis, neuropathology, temporal lobe epilepsy

## Abstract

Hippocampal sclerosis (HS) is a common pathology encountered in mesial temporal lobe epilepsy (MTLE) as well as other epilepsy syndromes and in both surgical and *post-mortem* practice. The 2013 International League Against Epilepsy (ILAE) classification segregates HS into typical (type 1) and atypical (type 2 and 3) groups, based on the histological patterns of subfield neuronal loss and gliosis. In addition, granule cell reorganization and alterations of interneuronal populations, neuropeptide fibre networks and mossy fibre sprouting are distinctive features of HS associated with epilepsies; they can be useful diagnostic aids to discriminate from other causes of HS, as well as highlighting potential mechanisms of hippocampal epileptogenesis. The cause of HS remains elusive and may be multifactorial; the contribution of febrile seizures, genetic susceptibility, inflammatory and neurodevelopmental factors are discussed. *Post-mortem* based research in HS, as an addition to studies on surgical samples, has the added advantage of enabling the study of the wider network changes associated with HS, the long-term effects of epilepsy on the pathology and associated comorbidities. It is likely that HS is heterogeneous in aspects of its cause, epileptogenetic mechanisms, network alterations and response to medical and surgical treatments. Future neuropathological studies will contribute to better recognition and understanding of these clinical and patho-aetiological subtypes of HS.

## Introduction

The hippocampus is the most widely studied brain region in both human and experimental epilepsy. Sclerosis of the hippocampus in epilepsy was first noted as far back as 1825 1 followed by the first detailed study of the segmental patterns of neuronal loss in a series of 90 *post-mortem* (PM) cases, published by Sommer in 1880 (2, for historical review see 3). In the modern era of epilepsy surgical programmes for the management of drug-resistant epilepsy, hippocampal sclerosis (HS) is one of the commonest pathologies 4. HS is particularly associated with the syndrome of mesial temporal lobe epilepsy (MTLE) but can be seen at PM in other epilepsy syndromes. It's incidence in large epilepsy surgical series varies from 33.6% 5 to 66% 6 and in PM series, HS is identified in between 30.5% and 45% of all epilepsy syndromes and in 56% with the syndrome of MTLE 7,8. Neuropathology research in MTLE/HS has been based largely on human surgical tissues, often in parallel with observations in the numerous experimental models of temporal lobe epilepsy (TLE). It has been directed into causes of HS, the cellular and molecular alterations that render the hippocampus epileptogenic, the identification of biomarkers that could be predictive of outcome following surgery as well as correlation with other comorbidities associated with seizures. This review aims to summarize some of the recent advances in this field which may be of relevance to the diagnostic neuropathologist.

## HS criteria, classification and patterns

There have been several schemes to classify subtypes of HS, based on the subfield distribution as well as extent of, hippocampal neuronal loss and gliosis. Other terms used interchangeably for HS include Ammon's Horn sclerosis (strictly refers to neuronal loss in CA1–4 and not the dentate gyrus) and mesial temporal sclerosis (which implies more extended sclerosis of extrahippocampal tissues, such as the amygdala and parahippocampal gyrus). A recent consensus classification system, validated through the neuropathology taskforce of the International League Against Epilepsy (ILAE) 9, aims to incorporate aspects of all previous schemes 10–15 (Table 1), through implementing a reproducible, semiquantitative scale for hippocampal subfield neuronal loss. The main advantages of the ILAE system is that it is based on standard stains (Table 2), so can be universally adopted in any centre, it clearly segregates ‘atypical’ (type 2 and 3) from ‘classical’ HS (type 1) and will reduce over-interpretation of endplate gliosis alone as HS. This new classification relies on patterns of neuronal loss and gliosis as objective measures of sclerosis and does not incorporate other alterations (e.g. mossy fibre sprouting, interneuronal alterations – see below) which may be more difficult to reproduce between laboratories. The obvious benefits of a single system is that it will enable comparisons of data sets between centres and emergence of accurate pathological and electroclinical correlations as well as with advanced magnetic resonance imaging (MRI) sequences 19. At present, for example, there is some evidence that HS patterns could be predictive of seizure history or outcome (as detailed in Table 2); the atypical HS patterns have been associated with poorer seizure-free outcomes 11. There is no evidence to support the proposal that type 2 and 3 evolve to type 1 HS over time. Implementation of this clear framework over the next years, integrated with clinical, genetic and imaging data will enable these issues to be addressed and clearer characterization of different subtypes of medial TLE with distinct aetiologies, network alterations and prognosis, in particular the likelihood of a long-term seizure-free outcome following surgical removal 20.

**Table 1:**
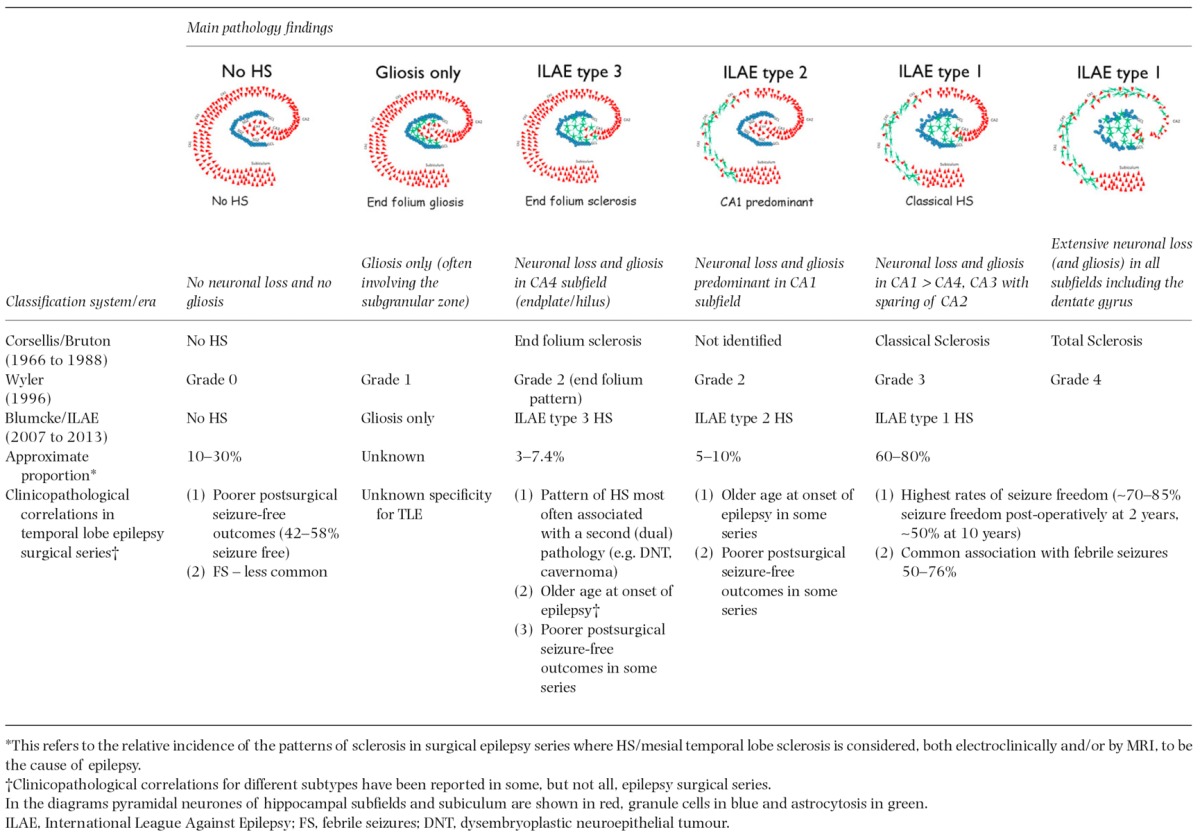
Summary of the various classification schemes adopted for the patterns of hippocampal sclerosis (HS) in epilepsy in recent eras

**Figure 1:**
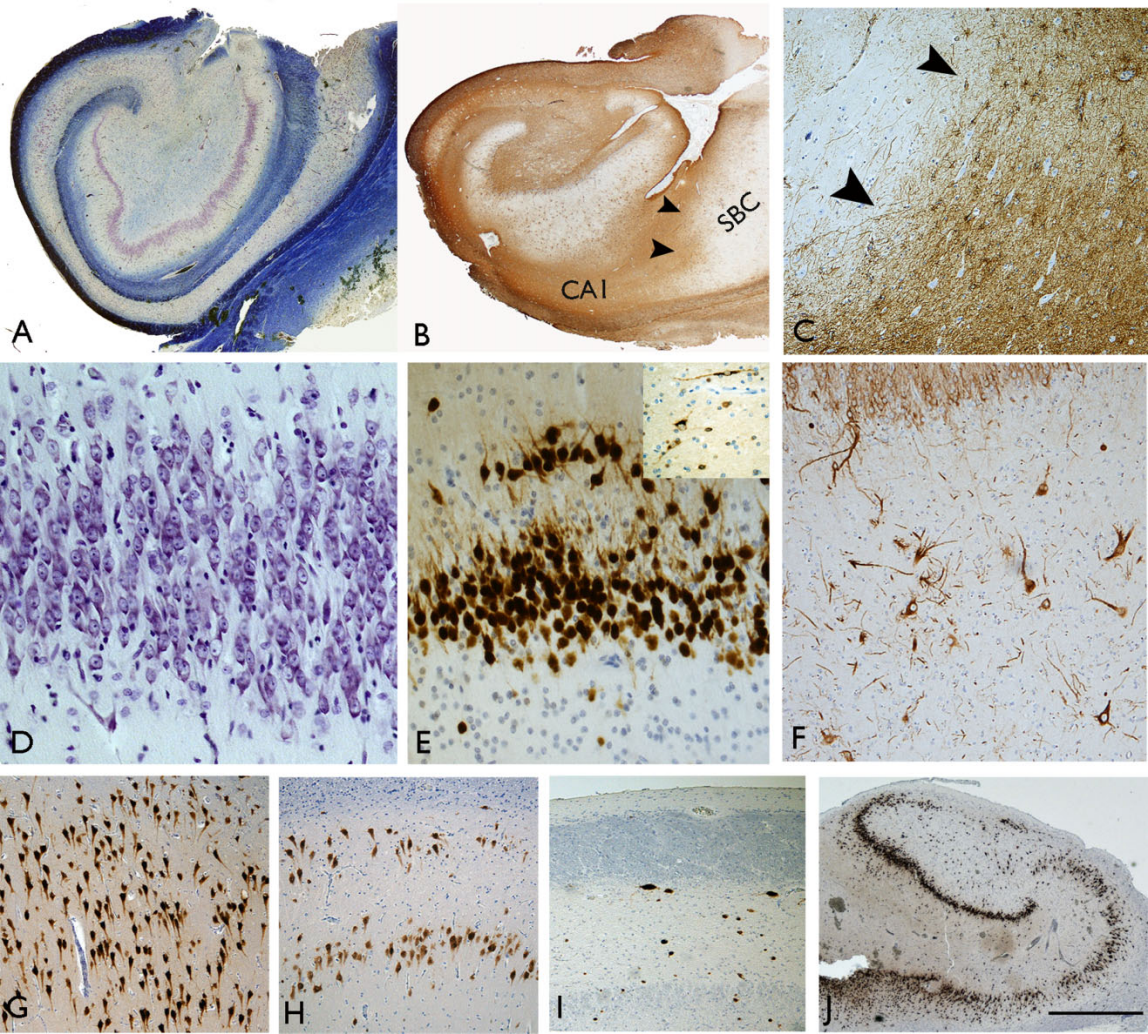
Typical pathology features of hippocampal sclerosis in epilepsy. (A) Luxol fast blue/cresyl violet-stained preparation of a surgical sample with subfield neuronal loss involving CA1 and CA4. (B) GFAP preparation confirming dense fibrillary gliosis in CA1 with a sharp cut-off point (arrows) with the adjacent subiculum (SBC) and (C) higher magnification detail of the abrupt transition of gliosis at the CA1/subiculum (arrows). (D) Granule cell dispersion as seen with cresyl violet and (E) with NeuN showing clusters of dispersed granule cells (inset shows reelin-positive interneurones and bipolar cells in the molecular layer). (F) Neurofilament-positive neurones with enlarged cell bodies in CA4 of the sclerotic hippocampus. (G) CA1 pyramidal cell layer in a patient with MTLE but no evidence of neuronal loss, (H) CA1 with evidence of partial neuronal loss from mid zone and, (I) CA1 with severe neuronal depletion and collapse of the layer. (J) Section of the pes hippocampus from a patients with confirmed classical ILAE type 1 sclerosis in the body; in the pes neuronal loss is limited to the endplate in this case as an illustration of variability in the pattern of atrophy that may occur along the longitudinal hippocampal axis. Bar is equivalent to approximately 1 mm (A, B, J), 250 micrometers (C, F, G, H, I) and 100 micrometers (D, E).

The histological hallmarks of HS have long been recognized since the earliest PM and surgical series 2,21. Distinctive histological features, which may aid in the discrimination from other causes of HS (e.g. neurodegeneration or hypoxia-ischaemia) include the sharp cut-off between the sclerotic CA1 and the spared neurones of the subiculum (Figure 1**A–C**), the typical fibrous, contracted gliosis of CA1 (attesting to the chronicity), granule cell dispersion (GCD) (Figure 1**D,E**), and scattered hypertrophic neurones (particularly in CA4) (Figure 1**F**) with cytoplasmic distention by microtubules and neurofilaments and increased dendritic complexity 22,23 (see Table 3). The diagnostic confirmation of HS relies to some extent on the provision by the surgeon of ‘en-bloc’ resections where the hippocampal subfield continuity is intact. In small, fragmented or poorly orientated specimens, confirmation of HS can be challenging 19, but supported through implementation of a small panel of special stains (Table 2). In anterior temporal lobectomy surgical procedures, an average 2 cm of the hippocampal body is resected 27,28. The pattern of sclerosis tends to be uniform along the longitudinal axis within a single surgical specimen in MTLE/HS 18 although variability, in the extent (Figure 1**G–J**) or distribution of subfield neuronal loss is occasionally seen; for example the pattern may represent type 1 HS at one coronal level and type 2 HS in another. The clinical significance of patchy *vs.* complete subfield damage in resected specimens is, as yet, uncertain 9.

**Table 2:** Panel of stains useful in the interpretation of HS in epilepsy; those shown in bold are used in the 2013 ILAE classification of HS 9

Histological preparation	Application	Methods/technical tips, limitation and pitfalls
**Luxol fast blue/cresyl violet**	Assessment of neuronal loss; these stains may be used interchangeably. NeuN is preferable for assessment of GCD. Neurofilament can highlight additional neuronal hypertrophy in CA4. Synaptophysin has also been used in the assessment of MFS, but it is not specific for mossy fibre pathway	NeuN is fixation sensitive and gives less consistent staining in *post-mortem* tissues* Thicker sections (>10 μm ) also recommended for assessment of cyto-architecture with NeuN
**NeuN**		
Synaptophysin				
Map2				
Neurofilament				
**GFAP**	Assessment of gliosis, patterns and distribution	Over-interpretation of endplate gliosis as sclerosis
GFAP-delta				
CD34				
Timm method 16	Assessment of mossy fibre re-organization	Timm stain: requires fixation of hippocampal slice from fresh specimen in buffered 1.2% sodium sulphide solution
Dynorphin ZnT3		Dynorphin: Thicker sections (>10 μm) recommended for better visualization of MFS
Parvalbumin	Assessment of interneuronal groups. Antibody clones used in illustrations in current paper: calbindin D-28K (1:10 000, Swant, Switzerland ) Calretinin (polyclonal, 1:2000; Sigma, Saint Louis, MO, USA) NPY ( 1:4000, Sigma) Parvalbumin, (1:300 Swant, Switzerland)	Parvalbumin can be fixation sensitive and give less consistent staining in *post-mortem* tissues*
Calbindin				
Calretinin				
Neuropeptide Y (NPY)				

^*^In *post-mortem* tissues, some markers may be fixation sensitive, for example parvalbumin, NeuN, requiring modified pretreatments 17; for Timm method in surgical tissues see 18.

HS, hippocampal sclerosis; GCD, granule cell dispersion; MFS, mossy fibre sprouting; ZnT3, zinc transporter 3.

## Granule cell dispersion (GCD)

Although there is no agreed definition for GCD in HS (granule cell layer thickness >10 cells 29 or 120 μm 30 have both been proposed), it is a common and striking feature in 40–50% of cases 29,31. The extent, as well as pattern of GCD, including ‘bi-laminar’ patterns or clusters of granule cells in the molecular layer (Figure 1**E**), may vary within cases and alternate with regions of granule cell loss. Extensive GCD is virtually pathognomonic of seizure-induced hippocampal changes and is typically seen in the context of hippocampal neuronal loss, particularly of CA4 13,32, suggesting that is an acquired process rather than a pre-existing abnormality. There is evidence linking the presence of GCD with early onset of epilepsy and febrile seizures (<4 years) as well as longer duration of epilepsy 31. There is conflicting data regarding whether GCD signifies a good outcome following surgery 13,31,32.There has been considerable interest in granule cell reorganization in HS from the perspective of its potential contribution to pro-epileptogenic circuitry, reflection of interference with ongoing neurogenesis and correlation with memory disorders associated with epilepsy.

Neuronal migration during development is normally associated with immature neurones. There are two schools of thought in HS: that GCD represents neuronal ‘heterotopia’ of newly generated neurones following aberrant neurogenesis or that it results from abnormal migration of mature neurones, both influenced by seizures. There is abundant evidence from experimental models that seizures influence rates of granule cell neurogenesis, with new neurones migrating to abnormal or ectopic positions and furthermore integrating into networks and acquiring pro-epileptogenic physiological properties 33–38. A model of febrile seizures recently reported that altered migration of newly generated granule cells was enhanced by excitatory gamma-aminobutyric acid (GABA) signalling during seizures 39. In contrast, following kainic acid-induced seizures, time-lapse studies argue for dispersion of fully differentiated granule cells 40, migrating by a process of ‘somatic translocation’, a mechanism which involves shifting of the cell body into an apical dendrite 41.

**Figure 2:**
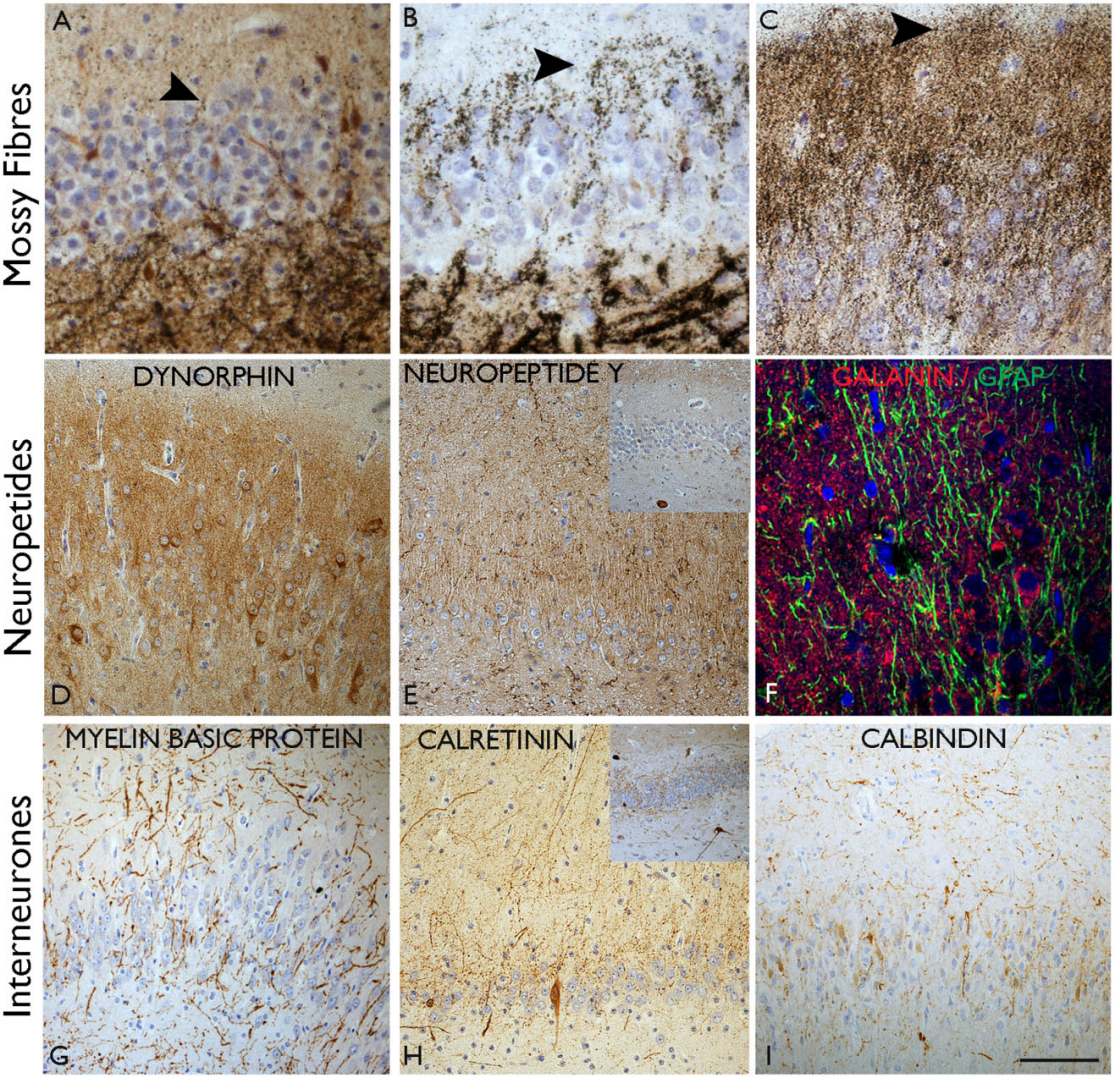
Axonal sprouting in hippocampal sclerosis (HS)/temporal lobe epilepsy (TLE). Mossy fibre sprouting identified with Timm staining in hippocampal sclerosis (HS) (A–C). In (A) the black silver granules are mainly confined to the subgranular zone and significant sprouting into the molecular layer (arrow) is not observed. In (B) there is focal sprouting in the molecular layer (arrow) and in (C) marked sprouting is shown with a dense band of zinc positive granules in the molecular layer. (D) Similar mossy fibre sprouting confirmed with dynorphin immunohistochemistry. (E) Extensive sprouting of neuropeptide Y-positive fibres is seen in the dentate gyrus compared with normal pattern (inset). (F) Sprouting of galanin fibres is shown in the gliotic dentate gyrus from a case with TLE and hippocampal sclerosis. In HS more intense synaptic pattern of staining was noted in the molecular layer of the dentate gyrus in a series compared with mTLE cases without HS (M. Thom, unpub. obs.). (G) The myeloarchitecture can appear abnormal in HS with a random arrangement of fibres permeating the dentate gyrus. (H) Calretinin immunohistochemistry with sprouting fibres through the molecular layer compared with the compact alignment of fibres embracing the dentate gyrus in the normal hippocampus (shown in inset). (I) Calbindin immunohistochemistry with sprouted fibres through the molecular layer in HS and loss of the normal expression in the granule cells. In all images the molecular layer is at the top of each figure and the subgranular zone at the bottom. Bar is equivalent to approximately 50 μm (A, B, C) and 100 μm in others.

It has proven more difficult to explore and validate any altered rates of neurogenesis or the migratory mechanisms that have contributed to GCD in TLE at the ‘end stage’ of the disease process, in either surgical or PM tissues 42–44. Morphological studies of dispersed cells in HS tissues have confirmed wider branching angles of the apical dendrites and more frequent recurrent basal dendrites, which could support that somatic translocation had occurred 45. Local deficiency of reelin protein and loss of reelin-expressing cells has been implicated as orchestrating the process of GCD (Figure 1**E**) 40,46,47. Studies of calbindin expression patterns, a marker of granule cell maturity, have also demonstrated differential expression in the dentate gyrus in HS with more dispersed cells calbindin positive, while more basal cells are calbindin negative; one interpretation of this is that a neo-migration of mature granule cell neurones has occurred 48–52 (Figure 2**A–C**). Important clinicopathological correlations include that a reduction of calbindin-positive granule cells 53, loss of granule cells in the internal limb of the dentate gyrus 54 as well as loss of regenerative capacity have all been associated with memory impairment arising in association with TLE 55. This highlights the important contribution of granule cell pathology to comorbidities in epilepsy.

## Neuropathology studies of epileptogenic processes in HS

The term *epileptogenesis* encompasses the cascade of cellular events, following which a brain develops spontaneous seizures or epilepsy. There are obvious limitations and challenges in exploring these processes in human tissues of HS, usually at an advanced stage and subjected to the effects of anti-epileptic drugs. The obvious question has always been how a region with such dramatic depletion of neurones can generate seizure activity. It is plausible that the pro-epileptogenic pathophysiological mechanisms are separate from the sclerosing process. Nevertheless, there has been a valuable contribution from neuropathological studies of altered neuronal networks and connectivity that could underpin hippocampal hyper-excitability to parallel observed altered neuronal electrophysiological properties in slice cultures 56. Furthermore, unravelling the key epileptogenic events is an essential pathway towards identifying novel molecular therapeutic targets to intervene with these processes 57,58.

### Mossy fibre sprouting

Axonal sprouting, a common process in the developing brain, is revived in adult tissues in response to seizures. Such plasticity may represent primarily a reparative response to hippocampal neuronal loss, but may ultimately be mal-adaptive and pro-epileptogenic 59. The mossy fibre pathway has been most widely studied in this respect. First observed in animal models, and subsequently in human HS 60, mossy fibre sprouting has been argued a critical component in the development of recurrent seizures in HS. In normal conditions, fewer than 1% of mossy fibres possess a recurrent axonal branch into the molecular layer but, in HS, extensive recurrent projection of mossy fibre collaterals into the molecular layer of the dentate gyrus occurs, to make excitatory synaptic contact 61 with apical dendrites and spines of granule cells in the inner molecular layer, essentially creating a local ‘short-circuit’ with a potential to synchronize neuronal groups. In tissues, mossy fibre sprouting is best demonstrated with Timm silver method (reaction with zinc, sequestered in synaptic vesicles) (Figure 2**A–C**) (Table 2), zinc transporter 3 16,62 or with immunohistochemistry for dynorphin A (an opioid neuropeptide present in granule cells and in the terminal fields of the mossy fibres) 63 (Figure 2**D**).

The extent of mossy fibre sprouting can vary between HS cases (Figure 2**A–D**). The process of sprouting is thought to be triggered early by both hippocampal seizure activity 37,64,65 and neuronal loss. There is some evidence that newly generated cells form a significant contribution to mossy fibre sprouting 36,52. Of note, it is absent or at the most mild in HS associated with neurodegeneration and dementia 24. In TLE patients with milder degrees of CA4 neuronal loss, mossy fibre sprouting may be less pronounced 66–68 as well as in HS with marked granule cell depletion. The molecular cues initiating or promoting sprouting include a role for mammalian target of rapamycin (mTOR) pathway activation; it has been shown that rapamycin administration reduces both sprouting and seizures experimentally 69,70 offering a potential novel treatment. There is also experimental literature to support that suppression of mossy fibre sprouting, for example with cyclohexamide, does not ameliorate seizures 71,72. In addition we have noted that sprouting remains sustained in elderly patients at PM with HS, even with spontaneous cessation of seizure activity for many years 73. These observations argue somewhat against a critical role of sprouting in hippocampal epileptogenesis. Therefore, although mossy fibre sprouting is a hallmark pathological feature of HS in epilepsy, any critical physiological contribution underpinning seizure activity remains to be established.

### Interneuronal networks in HS

**Figure 3:**
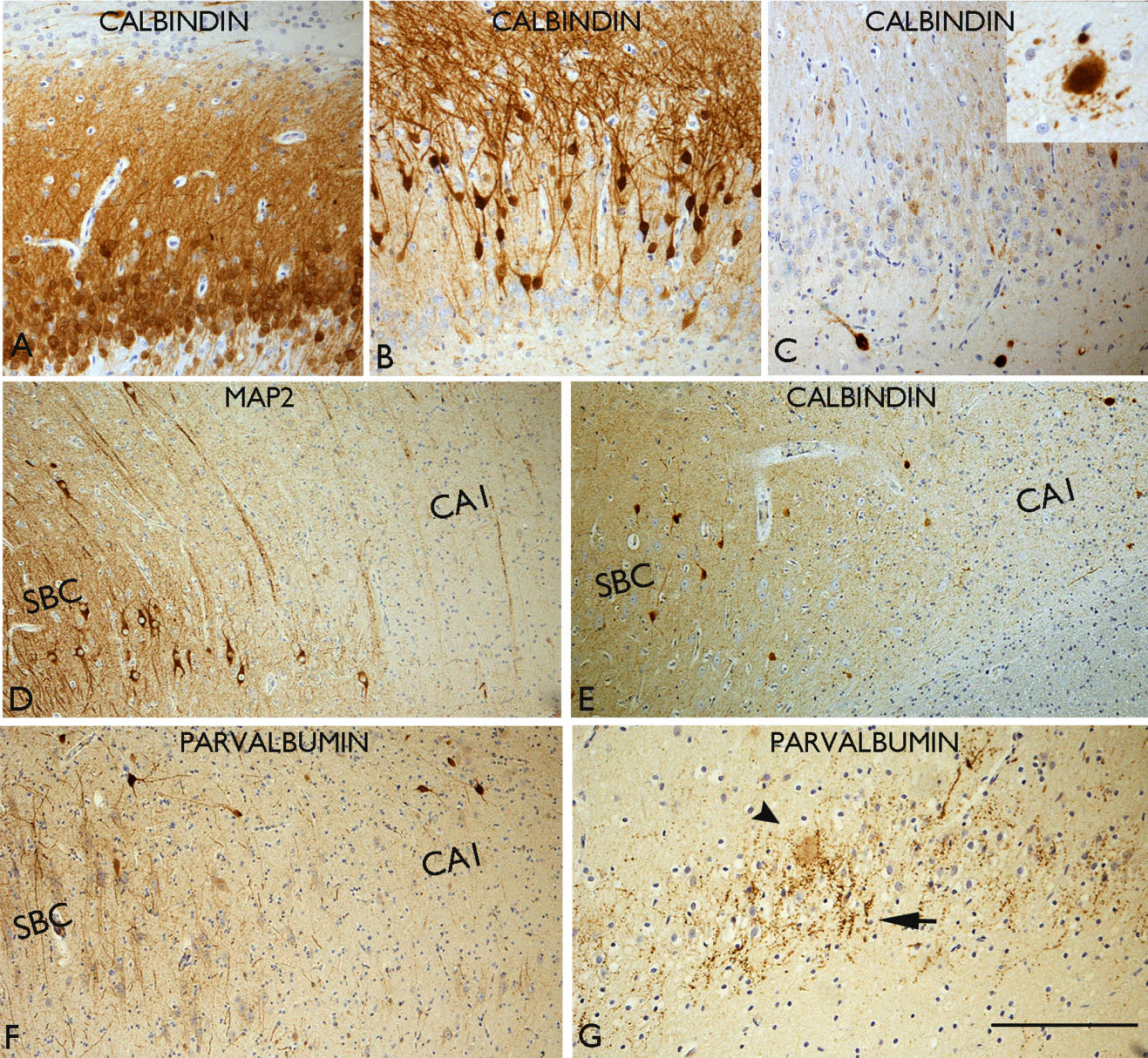
Interneuronal changes in hippocampal sclerosis in epilepsy (HS). (A) Normal calbindin pattern with labelling of the granule cells and apical dendrites. (B) Absent or loss of calbindin expression is noted in the basal cells while the dispersed cells are calbindin positive; this is a common pattern observed in HS with granule cell dispersion. (C) Virtual total loss of calbindin expression in all the granule cells; residual hilar calbindin-positive neurones as well as in CA1 (inset) can appear hypertrophic and dysmorphic with abnormal processes extending from the cell body. (D) The sharp transition between the neuronal loss in CA1 and the preserved subicular (SBC) neurones is illustrated with MAP2 staining. (E) Calbindin labelling in the same case as (D) shows relative loss of interneurones and their arborizations in the CA1 as with parvalbumin (F). Increased perisomatic labelling and terminals in the dentate gyrus, as with parvalbumin in an HS case. (G) Complex parvalbumin terminals are seen in the dentate gyrus granule cell layer in HS. Bar is equivalent to approximately 75 μm.

Although the ILAE classification of HS is based on patterns of principal pyramidal cell loss, stereotypical alterations to hippocampal interneuronal cell types also accompany this process. This has been studied more extensively in experimental models, where functional alterations in relation to cellular, network changes and epileptogenesis, can be directly correlated with histological changes to interneuronal groups 74. GABAergic cell types more extensively studied in human TLE/HS include those expressing calcium-binding proteins (calbindin, parvalbumin and calretinin) (Figures 2**H,I** and 3). Interneurones are also classified according to their morphology, localization and their target domain in the dentate gyrus and CA regions 75,76. In general they provide inhibition at the perisomatic or axon-initial segment of principle pyramidal neurones, for example parvalbumin-positive interneurones 50, or dendritic inhibition for single neurones or neuronal groups (including other interneurones), for example calretinin and neuropeptide Y (NPY)-expressing neurones 75,77. Both spatial and temporal variation in the vulnerabilities of specific interneuronal populations in response to seizures, is noted in experimental models 78.

The predominant qualitative and quantitative change reported in human HS studies include loss of protein expression or reduction in interneuronal number 50,79,80 (Figure 3**D–F**). Morphological changes include cell hypertrophy (Figure 3**C**), abnormal dendritic projections with altered distribution of spines 81–83 and axonal sprouting (Figure 2**E,F,G,H,I**). Axonal sprouting is the most readily demonstrated qualitative interneuronal change in routine diagnostic practice and can be a helpful aid, particularly in a fragmented HS specimen as well as in investigations of the aetiology of HS in PM samples 24,25,73 (Tables 2 and 3). It is exemplified in the dentate gyrus with calretinin, where expansion of the axonal plexus from the inner to the outer molecular layer may be observed (Figure 2**H**) 25, 81. Sprouting of calbindin-positive axonal networks through the dentate gyrus (Figure 2**L**) may also be evident in tissue sections, when unmasked by the loss of the normal calbindin expression in the dentate granule cells and dendrites, a common occurrence in HS (Figure 2**C**). Increased complexity of parvalbumin-positive chandelier cells and terminals in the pyramidal cell layer and the dentate gyrus has also been noted 50 (Figure 3**G**). Sprouting of these inhibitory networks is often observed to parallel sprouting of excitatory networks (the mossy fibres) in established HS 24,25,59,73. Many interneuronal alterations likely represent adaptive or compensatory responses to seizures but, nevertheless, may contribute to sustained excitatory/inhibitory imbalances as well as synchronization of local neuronal networks 74,84,85. As yet there is limited information regarding any differences in interneuronal changes that characterize ILAE HS subtypes. There is also experimental evidence, from stereological acquired data, that there is not necessarily a direct relationship between the extent of interneuronal loss and the severity of clinical seizures 86 which would also be important to further explore in MTLE subtypes in human tissues.

**Table 3:** Neuropathological features that may aid in the distinction of hippocampal sclerosis in epilepsy (HS-e) from other cause of HS 24–26

Histopathological features predictive of HS-e		
Highly associated HS-e	Not-predictive	Less likely HS-e
Dense fibrillary gliosis in CA1 CA4 neuronal loss Sharp demarcation between neuronal loss and gliosis in CA1 and the subiculum Granule cell dispersion Mossy fibre sprouting Interneuronal network reorganization Hypertrophic (NF-positive) neurones in CA4 (and other subfields)	CA2 sparing Neuronal loss and gliosis in CA1 is focal and not affecting all the subfield Variability of HS sclerosis along the longitudinal axis Bilateral HS Loss of calbindin expression in granule cells Microglial activation	Patchy, widespread cellular ‘reactive’ gliosis; less subfield restricted Neuronal loss in CA1 extends into subiculum Non-subfield specific distribution of neuronal loss Identification of neuronal inclusions with IHC (e.g. Tau, p62 and TDP43)

### Neuropeptides in HS

Neuropeptides colocalize with inhibitory neurones, are stored in large dense vesicles and when released, have longer half-lives than neurotransmitters, thus modulating neuronal or network activity over longer periods 87. NPY, somatostatin, galanin and dynorphin have endogenous anti-convulsant properties with protective effects against epilepsy, whereas substance P has a pro-epileptic effect 87. Many neuropeptides show high expression in hippocampal structures, particularly NPY 88. Experimental studies confirm release and increased synthesis of NPY and somatostatin following seizures 89–91. Reorganization of **NPY** fibre networks is the best studied and argued to ‘define’ the epileptogenic hippocampus 12,67,92. In the normal hippocampus, NPY-expressing cells are readily seen in the hilus of CA4 with a dense plexus of axons in the outer molecular layer, that synapse with granule cell dendrites 93 (Figure 2**E**). In HS, loss of NPY neurones and extensive sprouting and beading of NPY axons is noted, extending through the granule cell layer and into the molecular layer 67,92,94 (Figure 3**E**). This NPY-sprouting typically parallels mossy fibre sprouting and is thought to act by blocking the synchronization of granule cells through these recurrent mossy collaterals 95. NPY-sprouting has been reported in other (non-MTLE) epilepsy syndromes and in the absence of significant HS 73 but is significantly less pronounced, and often absent, in HS associated with dementia/neurodegeneration 24; as such it appears to represent a good histological marker for the epileptogenic hippocampus 95. Recently gene therapy studies using viral vectors to induce hippocampal NPY over-expression have resulted in decreased seizure frequency and remains an attractive treatment strategy 96. In addition, it has been recently recognized that NPY also regulates hippocampal neurogenesis, indicating roles beyond seizure modulation 97 but that may be relevant to cognitive decline in epilepsy.

Studies of **somatostatin**-expressing neurones in HS, similar to NPY, have demonstrated a reduction in cell number and radial sprouting of fibres in the dentate gyrus 67,92. **Galanin** is a small peptide of 29 amino acids with high concentrations in the adjacent amygdala 87. Its effects in the hippocampus are mediated by receptors GalR1 and GalR2 98 where it exerts a presynaptic inhibitory effect on glutamatergic transmission and is another promising treatment target 99. Alteration of galanin networks and receptor expression in human HS tissues, compared with other neuropeptides, remain less studied (Figure 2**F**). **Substance P** has been shown to have pro-epileptic effects with increased expression shown in status epilepticus 87. Studies of substance-P receptor expressing interneurones in HS have been carried out which have demonstrated a reduction of interneurones in CA1 and the hilus in addition to increased multipolar cells in the molecular layer; morphological changes were also noted including increased complexity of dendritic branches 81 and synaptic reorganization 100. These studies all reinforce the widespread alteration of neuropeptidergic systems in HS; as endogenous neuromodulators and a potential new therapeutic option, these systems, including the distribution of synthesizing neurones and their receptors, is likely to be more extensively studied in the future.

### Neurotransmitters in HS

**Figure 4:**
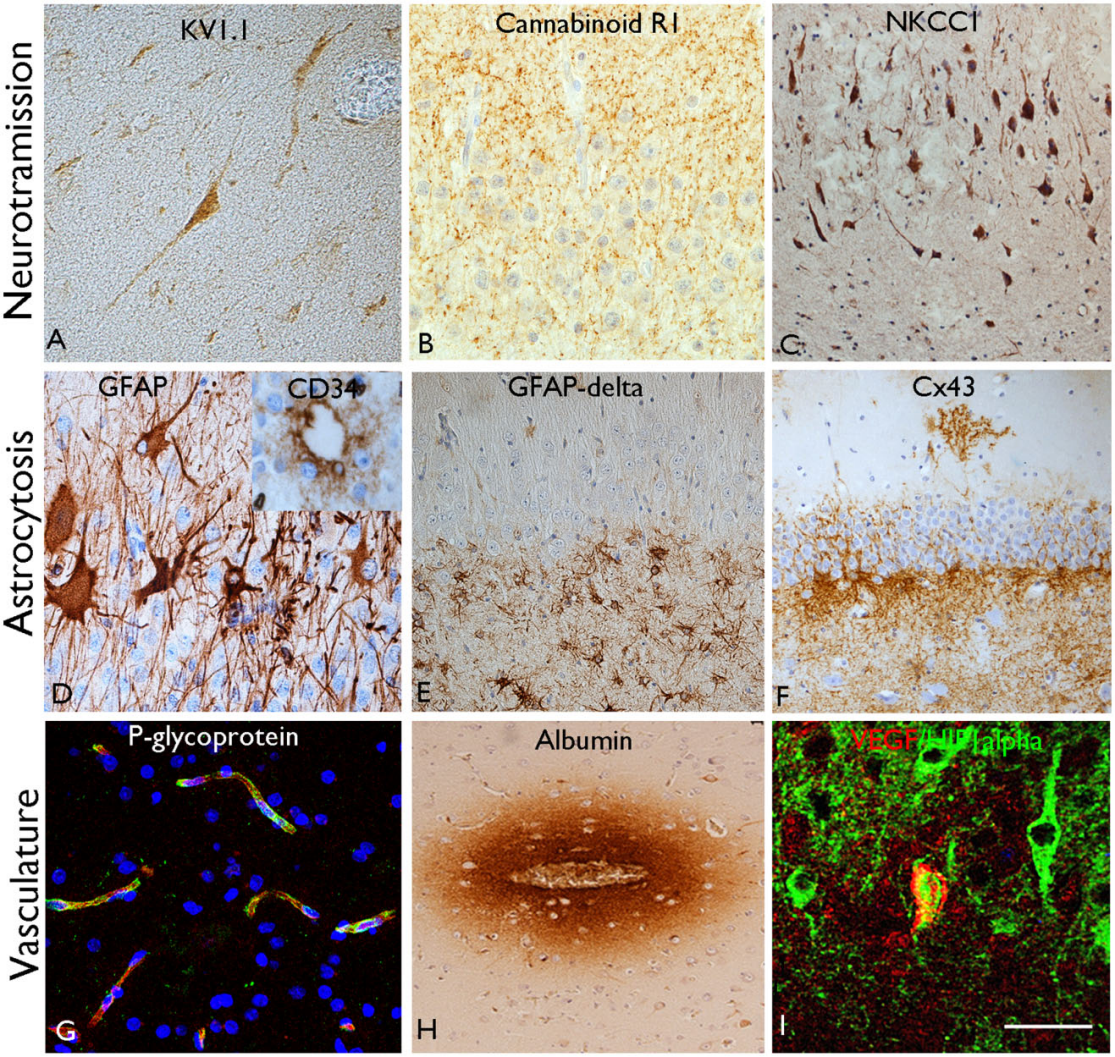
Pathological alterations potentially contributing to epileptogenesis in hippocampal sclerosis (HS). (A) Prominent labelling of voltage gate potassium channel (KV1.1) in residual neurones in CA1 in hippocampal sclerosis; differences were noted in the patterns of expression between hippocampal subfields in HS compared with controls (M. Thom, unpub. obs.). (B) Prominent labelling of cannabinoid receptor is noted in the plexus in the molecular layer of the dentate gyrus in HS. (C) Intense labelling of CA1 neurones with cation-chloride cotransporter (NKCC) in HS. (D) In some cases of HS in epilepsy, particularly in adult onset cases or in the context of recent encephalitis, striking reactive and ‘balloon cell’ gliosis can be seen in the granule cell layer with a proportion of these cells showing membranous CD34 staining, mimicking a focal cortical dysplasia (inset). (E) Immunolabelling with isoform GFAP-delta highlights prominent numbers of small astroglia, particularly in the subgranular zone, in HS. (F) Immunostaining for gap junction protein connexin 43 (Cx43) demonstrated prominent labelling of astrocytic cells in the subgranular zone and occasionally in the molecular layer in a *post-mortem* HS case. (G) Expression of drug transporter protein, p-glycoprotein, is shown on capillaries within hippocampus. (H) Albumin staining demonstrating focal leakage from small capillary vessels in a case of HS/TLE. (I) Coexpression of vascular endothelial growth factor (VEGF) and hypoxic inducible factor 1 alpha (HIF1-alpha) is shown in pyramidal neurones in HS. Bar is equivalent to approximately 35 μm (A, B, D, E, I); 60 μm (C, E, F, H).

Abnormalities in the regulation of synaptic neurotransmission in HS can occur at any level from protein synthesis, release, to receptor distribution and assembly and subsequent signalling cascades 101. Monogenic epilepsy syndromes commonly involve mutations in ion channel or neurotransmitter receptor proteins 102. The genetics of partial epilepsies, including TLE are likely to be complex 103; familial forms of TLE with HS are recognized and some of the candidate genes also involve ion channels 104 as well as in sporadic TLE 105. For example, a recent genome-wide association study has linked a sodium channel gene cluster of SCN1a in patients with HS and a history of febrile seizures 106. On this theme, abnormalities in the expression and distribution of voltage-dependent potassium channels have been shown in tissue sections in HS 107 (Figure 4**A**). Altered GABA_A_ neurotransmitter receptor expression, distribution and assembly has also been reported in HS; these mediate fast postsynaptic inhibition 108, and alterations are particularly noted in the dentate gyrus where the granule cells display remarkable plasticity 109,110. Extracellular GABA concentration is influenced by the rate of uptake of this neurotransmitter and altered levels of GABA transporters GAT-1 and GAT-3 have been shown in TLE 111. The endocannabinoid system also modulates glutamatergic and GABAergic synaptic transmission 112 with a proposed role for seizure potentiation in TLE 113. In tissue sections of HS, a decrease in cannabinoid type 1 receptor (CB1) and CB1 receptor-binding protein mRNA has been observed 114 but with increased levels associated with GABAergic fibres in the dentate gyrus 115, suggesting a primary modulation of GABAergic transmission (Figure 4**B**). There is also evidence for a switch from an inhibitory to excitatory action of GABA in epilepsy 116,117, mirroring its developmental function. This change is mediated by altered expression of neuronal cation-chloride cotransporters (KCC2 and NKCC1) 118. Changes in the relative expression of NKCC1 and KCC2 in pyramidal cells of hippocampal subfields has been shown in TLE/HS (Figure 4**C**) which may contribute to epileptiform activity 119,120.

### Astroglia and blood brain barrier dysfunction

Gliosis is a striking component of HS with chronic, fibrillary gliosis in CA1 and a radial gliosis in the dentate gyrus 43; gliosis involving CA4 is a less specific finding for HS, being a common finding in the absence of neuronal loss. A few case reports of HS have also noted a more cellular gliosis with CD34-positive ‘balloon cell’ like astrocytes in the dentate gyrus, reminiscent of those observed in focal cortical dysplasia (Figure 4**D**). These astroglia were associated with striking rarefaction of the dentate hilus 121 and were also reported arising in HS following a nonherpetic limbic encephalitis 122 and may indicate a different aetiology of the HS in epilepsy. Glial fibrillary acidic protein (GFAP)-delta, a developmentally regulated isoform, highlights small multinucleate glial cells, particularly in the subgranular zone of the dentate gyrus which colocalize with nestin in HS (Figure 4**E**) and may represent a specific subpopulation of glia with specific roles in this neurogenic niche 123; similar glial cell types were also noted in HS associated with dementia 24.

Over the last decade there has been a trend away from a ‘neurocentric’ focus with more directed investigations addressing the participation of astrocytes in the causation of seizures 124. There is a breadth of astrocytic function beyond their acting as supporting cells 125; dysfunctions of many of these processes may be of relevance to seizures in HS. Evidence for this includes impaired glutamate clearance by astrocytes and release in HS (via EAAT1 and EAAT and glutamine synthetase) 126 and impaired K+ clearance via inwardly rectifying potassium channels 127. Astrocytes are extensively coupled via gap junctions to form a functional syncytium, primarily expressing connexin 43. There have been conflicting findings, however, regarding the elevation of these proteins in hippocampal astrocytes in HS 127–129 (Figure 4**F**).

Abnormalities of the vasculature have been reported in HS, with proliferation of micro-vessels 130, vascular endothelial growth factor receptor expression and loss of blood-brain barrier integrity 131,132. Vascular leakage of proteins, including IgG 133 and albumin may contribute to neuronal dysfunction in epilepsy 134 (Figure 4**H**). Of note, other studies, demonstrated a reduced microvasculature in the sclerotic hippocampus 135,136. Over-expression of drug transporter proteins at the blood brain barrier in HS, such as p-glycoprotein and multidrug resistance-associated proteins, have been linked to treatment failure in HS, due to facilitated drug efflux preventing anti-epileptic drugs from reaching their target neurones 137,138 (Figure 4**G**).

## Aetiology of HS

### Initiating insult and genetic susceptibility factors

The aetiology of HS is still controversial and is likely to be multifactorial. It is widely considered an acquired pathology. Alfred Meyer's seminal hypothesis from the 1950s proposed that an initiating event, injury or insult the initial precipitating injury’ (IPI), particularly prolonged febrile convulsions early in life, primed the immature hippocampus for the subsequent development of HS 139,140. Seizures are known to damage the hippocampus, particularly prolonged seizures and status epilepticus 141–145. Experimentally, TLE has been induced following prolonged febrile seizures 146 and prospective studies, as the FEBSTAT study have confirmed HS (as defined by MRI criteria) in a minority of patients following febrile status epilepticus 147. However, as neither status epilepticus, prolonged febrile seizures nor repetitive generalized or partial seizures inevitably lead to HS in the majority of cases, there are likely other susceptibility factors. HS is considered a sporadic condition. Rare pedigrees provide evidence for a common genetic basis for febrile seizures and MTLE 148–150. Although genetic susceptibility determinants to nonfamilial or sporadic HS have, as yet, not been clearly defined 103,151–153, ApoEε4 genotype has been associated with increased risk of bilateral HS 154 and more recently, HS and febrile seizures were linked by common genetic variation around *SCN1A* gene 106.

### Seizure-induced neuronal loss

Necrotic or apoptotic neurones are only very occasionally seen in surgical specimens of HS as evidence of ongoing neuronal death. It is generally regarded that seizure-induced neuronal injury and subsequent loss results from excitotoxic, glutamatergic neurotransmission, excessive Na+ and Ca^2+^, resulting in osmolytic stress and cellular free-radical production, culminating in necrosis of neurones 155. Molecular studies also support that activation of apoptotic cell death pathways (mediated by both intrinsic and extrinsic pathways) contribute to hippocampal neurone loss 155,156. Regarding the distribution of neuronal loss in HS, subfield specific regulation of microRNAs has been shown following seizures; these post-transcriptional regulators of gene expression may be critical for determining cell death pathways 157. Hypoxic inducible factor 1 alpha (HIF1-alpha) and vascular endothelial growth factor (VEGF) neuronal induction has been shown in HS cases at PM, with some evidence of correlation of seizure activity prior to death 158 (Figure 4**I**). HS can be observed in association with a second seizure-focus or epileptogenic pathology such as a low grade tumour/malformation, e.g. dysembryoplastic neuroepithelial tumour (DNT), cavernoma; the pattern of HS is often type 3 in such ‘dual pathologies’ 9 and mechanisms of this potentially ‘kindled’ hippocampal neuronal loss may differ from isolated HS. Indeed, in paediatric MTLE/HS series, a higher percentage of cases have dual pathologies compared with adult surgical series; this fact, together with hippocampal maturation, may be relevant to different patterns of neuronal loss and dentate gyrus abnormalities observed between age groups 159,160. Mitochondrial dysfunction has also been proposed to play a role in the underlying pathogenesis of HS 161.

### Inflammation

In HS, neuronophagia or focal infiltrates of microglia are occasional findings. There is evidence to support activation of both the innate and adaptive immune system, for example IL-1β and IL-1 receptor upregulation, has been noted in astrocytes, microglia and neurones in HS, and intercellular adhesion molecule 1 (ICAM-1) and kallikrein expression in glia 162,163. B and T cell infiltrates, however, are usually inconspicuous in tissue sections and mainly in a perivascular location 162–165. Inflammatory pathway activation in human TLE is supported by gene expression studies 166 and has been argued to be a driving force in disease progression 167. There is evidence that inflammation can perpetuate, augment or even initiate seizures 168,169. A potential diagnostic pitfall to consider is that localized active/chronic inflammation can be seen in the hippocampus in relation to prior invasive depth electrode recordings. However, the presence of prominent, more widespread inflammation in HS specimens, nevertheless, should always raise the possibility of an underlying or previous limbic or autoimmune encephalitis, particularly in adult onset epilepsy cases 170. Regarding underlying viral infection in HS, detection of human Herpes virus 6 infection was frequently reported in one HS/TLE series 171 but in another was only identified in patients with a history of a prior episode of limbic encephalitis 172.

### Underlying maldevelopmental template

Hippocampal sclerosis can be observed in association with generalized or focal malformations of cortical development (MCD) as a dual pathology 173 and with focal cortical dysplasia (FCD) Type IIIa of the temporal lobe 174. There has been ongoing debate regarding if a pre-existing isolated hippocampal developmental abnormality could precede HS, and predispose to the development of sclerosis 29,175–178. A recent MRI study of family members of patients with TLE and HS confirmed smaller hippocampal volumes than normal, which may represent a developmental variant at risk for HS 179. Indeed recent genome-wide studies have also linked common genetic variants which associate with hippocampal volume 180,181; further study may identify how these genes influence hippocampal development and if they are of relevance to loss of hippocampal volume and vulnerability to HS in epilepsy. There is also ongoing debate regarding the relevance of hippocampal malrotation (also known as incomplete hippocampal inversion) to epilepsy, TLE, HS and febrile seizures 182. In malrotation, the pyramidal cell layer of the CA1/subiculum region typically appears preserved but hyper-convoluted. There is no evidence to support that this necessarily evolves into the typical picture of HS in patients with epilepsy 183,184 and there is also a lack of the mossy fibre sprouting, GCD and interneuronal re-organization that typify HS 175,183,185.

## Studies of HS in PM tissues

**Figure 5:**
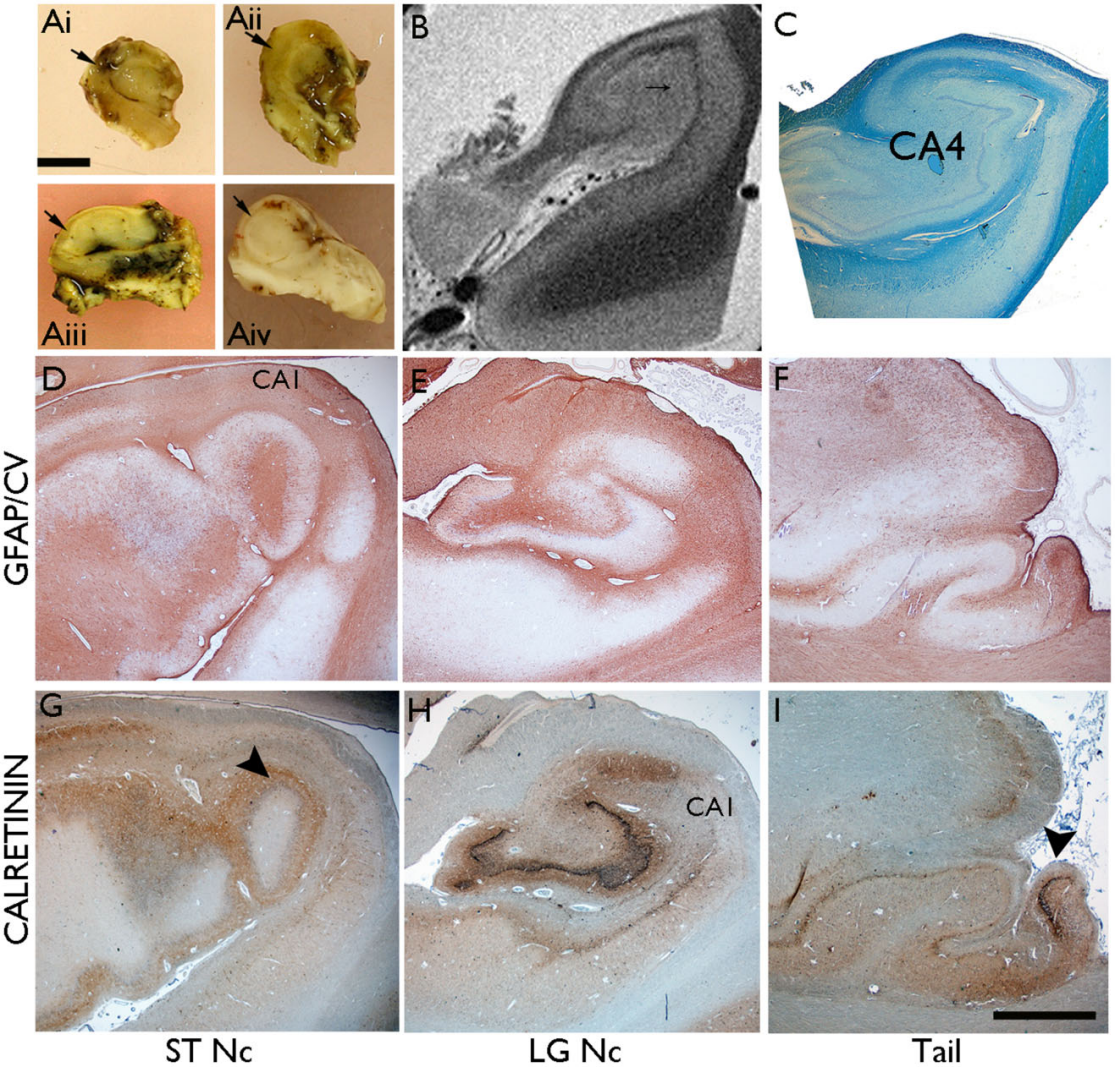
Variability of hippocampal sclerosis (HS) in *post-mortem* and surgical samples. (A) Surgical hippocampectomy specimens, which on histological examination correlated to (i) end folium gliosis with no evidence of sclerosis; (ii) ILAE Type 3 HS (end-folium sclerosis); (iii) ILAE type 2 HS (CA1 predominant sclerosis); and (iv) ILAE type 1 HS (classical hippocampal sclerosis). In all of the images the arrow indicates the pyramidal cell layer of CA1. (B) A 9.4T MRI image of a *post-mortem* hippocampus from a patient with longstanding epilepsy and ILAE type 2 (CA1 predominant pattern) of HS at this level (shown in C in a luxol fast blue/cresyl violet preparation), although in other levels the pattern was type 1 (classical). The MRI has the ability to identify subfields and white matter tracts and, indistinctly (arrowed), the dentate gyrus. Improved high fields sequences in the future may be able to identify and define patterns of HS pre-operatively. The MRI image was provided with courtesy of Dr Sofia Eriksson at the Department of Clinical and Experimental Epilepsy, UCL, Institute of Neurology. (D to I) Paired sections of hippocampus from one hemisphere labelled with (D to F) GFAP/counterstained with cresyl violet and (G to I) calretinin: at the level of the subthalamic nucleus (STNc; D, G), lateral geniculate nucleus (LGNc; E, H) and hippocampal tail (F, I). Classical pattern (ILAE type 1) HS is seen in the anterior levels with sprouting of calretinin-positive fibres visible in the dentate gyrus (arrow) at this low magnification; in the tail gliosis and neuronal loss is visible in the CA4 region of the hippocampal tail. Bar is equivalent to approximately 3 mm (D to I).

Although the bulk of neuropathology research in the past decades has been carried out on surgical tissue with its obvious advantages optimally preserved tissues, homogeneous clinical cohorts and access to up-to-date electroencephalography (EEG) and neuroimaging studies we should not neglect the ongoing contribution that autopsy samples can provide in the study of HS in epilepsy. Primarily, PM tissues enable comparisons of HS occurring in epilepsy syndromes other than TLE (also known as secondary HS 7), the effects of a lifetime of seizures on the severity of hippocampal neuronal loss and its associated pathology 186, enable study of the bilaterality of HS 73, the extent of involvement along the entire length of the hippocampus 25 (Figure 5) and to address degeneration in wider networks that have been implied from quantitative MRI studies in TLE 20,187,188, in particular the amygdala, cortex 189, thalamus 190 and cerebellum 191. Neuropathology studies have, from the outset, recognized that more extensive pathology may accompany HS 15,21, the hippocampus being the epicentre of a wider process. Experimental data support widespread changes occurring following induced seizures 192 which could equally contribute to epileptogenesis. Involvement of wide networks has been shown in TLE 193,194 and functional and structural imaging of TLE indicates altered brain connections or connectome in TLE 195,196. As such, in recent years there has been a move away from a ‘hippocampocentric’ view of TLE 197,198 addressing contribution from other brain regions. The main studies have investigated (i) electrophysiological evidence to support origin of seizures from extrahippocampal structures 199,200; (ii) if wider disease offers the explanation for poor outcomes (in terms of seizure-freedom) following localized surgery; and (iii) if severity (or progression) of any extrahippocampal pathology correlates with comorbidities, such as cognitive decline.

The cellular and pathological basis of structural changes in extrahippocampal regions associated with HS typically manifests as neuronal loss and gliosis with few studies exploring the contribution of interneuronal populations 189,201. It remains to be established whether any more extended ‘network’ changes arise as a result of the same initial insult causing HS, if they are subsequent to HS through retrograde/anterograde degeneration, or if they arise independently. In PM series it is also possible to explore the long-term effects of seizures on the brain and any predisposition to neurodegenerative disease or accelerated ageing processes in HS. It has been observed from a patient cohort closely followed for decades at the National Society for epilepsy, Chalfont centre, that patients with pathology proven HS/TLE at PM were less likely to go into terminal seizure remission with advanced age compared with other epilepsies 8. It has also been shown in this same PM cohort that accelerated tau accumulation in epilepsy was not associated with the severity of seizures and was also independent of the presence of HS but correlated with acquired and accumulative traumatic brain injury incurred from frequent seizures 202. Continued donation of brain tissues from patients with epilepsy to tissue bank resources for example the Epilepsy Society Brain and Tissue Bank at University College London (UCL) will enable further studies of HS in the future.

## Outcomes and the future

Although, benign forms of TLE and HS exist with infrequent and well controlled seizures 203 many patients with HS are medication-resistant and surgical intervention, following a series of investigations such as imaging, functional tests and video-EEG 204, may offer the best treatment option at present. Temporal lobe resection techniques have evolved, specifically with more limited resections, aiming to preserve function without compromising seizure outcome. Of the selected patients who undergo surgical treatments, approximately two-thirds will remain seizure-free in the first 2–3 years with around 57% seizure-free outcome at 5 years 6. The cause for surgical failure and poor outcomes in a proportion if carefully selected patients, is as yet, unclear.

In summary, there is accumulating evidence that HS in epilepsy is likely to be heterogenous in many aspects, including its aetiology, genetics, epigenetics, networks involved, patterns of neuronal loss as well as responses to drugs and surgical treatments 20. Neuropathology- and tissue-based studies in the future, are likely to continue to contribute to the ongoing identification of diagnostic and prognostic biomarkers as well as to further our understanding of the causes and ultimately, the prevention of this pathology.
